# Maternity Service in Bristol

**Published:** 1952-04

**Authors:** F. G. Mogg


Whilst sharing the enthusiasm of Professor Lennon for the provision of a first-class Maternity Service, I found his article disappointing in the inadequacy and impracticability of his suggestions.

The traditional relationship between doctor, patient and consultant has proved over a long period to be too valuable to all concerned to be lightly discarded. Competition between doctor and consultant for patients destroys this relationship utterly.

Professor Lennon wants all patients to be seen in a clinic at least twice during their pregnancy. The advantages of this are not clear. How can it benefit a patient to attend a hospital if she expects to be delivered elsewhere? Are not the hospital services of X-rays and blood test already available to the G.P.? Will the Consultant or his registrar who sees a patient in a clinic be the one to attend that patient in an emergency? Even if this is ensured, is it an important matter?

I agree with Professor Lennon that there are too many clinics: all should be closed except those held by Consultants who see patients referred to them, and those for teaching students and nurses. When many patients could not afford to be treated privately all these clinics were necessary, but the health service has altered all that. The clinics run by the Public Health authorities are invaluable in educating the patient; but they are expensive and are unnecessary for antenatal supervision. Moreover the staff often has had only limited midwifery experience.

I know that there are some G.P's who provide an indifferent service for their maternity cases. One of the causes of the rift between the midwife and the G.P. is the poor opinion the former has of the G.P. obstetrician who limits his attendances to the statutory two antenatals and one postnatal, and who tells the midwife not to send for him if the case comes off in the night, or even, not to send at all! Although it is not essential for doctors personally to deliver their cases, a visit from them during labour and one or two in the puerperium mean much to the patient.

Nowadays strange midwives commonly ask if one wants to be called, or not. The prevalence of this question points to a lack of interest on the part of some G.P's and lends weight to the claim that a midwife can deal with a normal confinement as well as a G.P., if not better. Those who make this claim insist that any abnormality should be dealt with by a full time obstetrician, and it is obvious that if this argument prevails the G.P. will eventually be debarred from midwifery. Some blame for this must fall on those practitioners who are bringing the G.P. obstetrician into disrepute, and are injuring the status of those doctors whose special interest and skill justifies their inclusion on the Obstetric List. It is this minority who cause Professor Lennon and many others who have the welfare of the patient at heart to hanker after that detestable word " supervision ".

Frank G. Mogg

